# Genetic Considerations in Psychiatric Treatment for Individuals with Intellectual and Developmental Disabilities (IDD)

**DOI:** 10.1192/j.eurpsy.2025.1555

**Published:** 2025-08-26

**Authors:** D. B. Valle, H. M. Nguyen, B. R. Carr

**Affiliations:** 1 College of Medicine, University of Florida, Gainesville, FL, United States

## Abstract

**Introduction:**

Genetic testing has become an essential tool in managing psychiatric conditions among individuals with intellectual and developmental disabilities (IDD). The frequent presence of genetic syndromes within this population underscores the value of genetic insights for guiding personalized treatment. By integrating pharmacogenetics into care, clinicians enhance medication efficacy and reducing the likelihood of adverse effects.

**Objectives:**

This study aims to elucidate the role of genetic testing in identifying specific syndromes and psychiatric comorbidities in IDD, explore the application of pharmacogenetic insights to optimize psychiatric medication management, and highlight case examples that demonstrate how genetic insights influence treatment decisions.

**Methods:**

A targeted literature review identified relevant studies on genetic syndromes linked to IDD, the impact of genetic testing in psychiatric care, and the application of pharmacogenetic data in guiding medication management. Key studies examined the pharmacogenetic profiles of individuals with IDD and their effects on treatment outcomes, focusing on findings that can inform personalized treatment strategies.

**Results:**

Fragile X syndrome (FXS), a prevalent genetic disorder among IDD populations, is associated with high rates of anxiety, ADHD, and autism spectrum disorder (ASD). Research shows that stimulant medications commonly used for ADHD may worsen agitation in individuals with FXS, making non-stimulant options or behavioral therapies preferable. Pharmacogenetic findings, such as variations in serotonin-related gene polymorphisms, have been linked to increased aggression and stereotypic behaviors in males with FXS, guiding clinicians toward selective serotonin reuptake inhibitors (SSRIs) or alternative behavioral approaches. In cases of DiGeorge syndrome (22q11.2 deletion syndrome), genetic testing identifies an increased risk for psychotic disorders, and genetic variants may affect drug metabolism. Findings from the IMPACT study, a Centre for Addiction and Mental Health (CAMH) project, highlight the benefits of tailoring medications based on genetic markers, such as CYP2D6 and CYP2C19 enzyme activity. These insights improve outcomes by reducing side effects like sedation and enhancing therapeutic efficacy, especially for IDD patients who may respond unpredictably to psychiatric medications.

**Conclusions:**

Genetic testing is invaluable in the psychiatric management of individuals with IDD, offering insights that enable more personalized and effective treatment approaches. By identifying genetic syndromes and incorporating pharmacogenetic data into medication planning, clinicians can minimize adverse effects, tailor therapies, and improve patient outcomes. Ongoing research and broader access to pharmacogenetic testing are essential to further refine treatment protocols for the IDD population.

**Disclosure of Interest:**

None Declared

